# Typification and Taxonomic Remarks on Names of *Iris* (Iridaceae) Associated with the Turkish Flora

**DOI:** 10.3390/plants10071486

**Published:** 2021-07-20

**Authors:** Eugeny V. Boltenkov, Adil Güner, Alexander A. Kuznetsov

**Affiliations:** 1 Botanical Garden-Institute, Far Eastern Branch, Russian Academy of Sciences, 690024 Vladivostok, Russia; 2 Nezahat Gökyiğit Botanic Garden, Ataşehir, İstanbul 34340, Turkey; adil@ngbb.org.tr; 3 Laboratory of Herbarium, Tomsk State University, 634050 Tomsk, Russia; ys.tsu@mail.ru

**Keywords:** Iris, epitype, lectotype, neotype, taxonomy, Turkish plants, typification

## Abstract

In view of the forthcoming review of Turkish irises for *Resimli Türkiye Florası* (*The Illustrated Flora of Turkey*), nineteen names are typified or nomenclatural remarks are provided in the present report. Lectotypes are designated for *Iris aschersonii*, *I. attica*, *I. bornmuelleri*, *I. purpureobractea* (a taxonomic synonym of *I. junonia*), *I. reticulata* var. *cyanea*, *I. reticulata* var. *sophenensis*, *I. suaveolens* and *I. taochia*. The second-step lectotypification is made for *I. histrio*. Neotypes are designated for the names *I. histrioides*, *I. junonia*, *I. masiae* and *I. reticulata* var. *histrioides*; epitype, for the name *I. reticulata* var. *sophenensis*. For the previously typified names, *I. bakeriana*, *I. musulmanica* and *I. reticulata*, lectotypes are given. The lectotypes for *I. histrio* var. *aintabensis*, *I. schachtii*, and *Xiphion danfordiae* and the authorship for *I. histrioides*, are corrected. Images are provided for eight specimens selected as types that are not available online. Notes on distribution in Turkey are provided for all the accepted taxa.

## 1. Introduction

*Iris* L. is a taxonomically challenging and the largest genus in Iridaceae with ca. 280 species, distributed mostly in the Northern Hemisphere [1]. In Turkey, it is represented by rhizomatous or bulbous perennials growing in various habitats. Boissier [2] mentioned a few *Iris* species from Turkey for the first time. From a taxonomic point of view, the study of Turkish irises of the section *Oncocyclus* (Siemssen) Baker and subgenera *Hermodactyloides* Spach and *Scorpiris* Spach was initiated by Güner and Peşmen [3]. Mathew [4] was the first to carry out a complete taxonomic overview of the *Iris* species from Turkey. According to Mathew, in Turkey, the genus comprises 37 species. The recent Checklist of the Turkish flora by Güner [5] includes 50 species.

Recently, many taxonomic studies on plant species are oriented towards typification in order to establish choices of type or to identify cases where corrections are necessary to avoid nomenclatural disruption (e.g., [6]). During the revision of the genus *Iris* for the forthcoming Volume 3 of *Resimli Türkiye Florasi* (*The Illustrated Flora of Turkey*) by the second author, it became evident that most Turkish iris names required typification. Besides, some species names had been cited incorrectly or with incorrect authorship [7,8]. This report aims to summarize information concerning the previously typified names and to select the types of the remaining untypified names associated with the Turkish flora.

## 2. Materials and Methods

This work is based on a comprehensive study of the relevant literature, in addition to the protologues. Herbarium specimens deposited at AEF, ANK, B, BAK, BEI, BM, E, G, HUB, ISTE, JE, K, KFTA, LD, LE, M, MHA, NGBB, P, TBI, TGM, W, WAG, and WU (herbarium codes according to Thiers [9]) were examined. The lectotypes, epitypes, and neotypes are designated here following the recommendations of the *International Code of Nomenclature for algae, fungi, and plants* [10] (ICN). The taxa are arranged in alphabetical order of the names under which they were originally described. The accepted names are highlighted in bold. The relevant information indicated in the protologue (“Protologue citation”) is provided here for all the names. Protologues and labels, originally composed in Russian and Turkish, were translated into English. Specimens were physically seen (!) unless indicated otherwise (i.e., [digital image!]). All specimens are cited in full, and most of them were assigned the barcode numbers following the herbarium acronyms. For the specimens deposited at AEF, HUB, ISTE, and TGM, inventory numbers are indicated. A conservative taxonomy of *Iris* is here used (e.g., [5,11,12]). For each accepted taxon, notes on distribution in Turkey are provided.

## 3. Results and Discussion

### 3.1. Typification of the Names

(1) ***Iris aschersonii*** Foster, Garden (London 1871–1927), 61(1): 288, 1902 ≡ *I. grant-duffii* var. *aschersonii* (Foster) Hayek, Ann. K. K. Naturhist. Hofmus. 28: 181, 1914 ≡ *Syrianthus aschersonii* (Foster) M.B.Crespo, Mart.-Azorín & Mavrodiev, Phytotaxa 232(1): 62, 2015.—“*Iris grant-duffii* subsp. *aschersonii* (Foster) Dykes”, Gen. *Iris*: 45, 1913, *nom. inval.* (see Art. 35.2, Ex. 6 of the ICN)—Protologue citation: “… Cilicia near Adana, Siehe”.—Lectotype (designated here): [Turkey, Adana Province] *Iris Aschersoni* Fost. & Siehe *n. sp.* Ebene Ciliciens Auf Kalk. b. Adana, 1898 aufgefunden und als neu erkannt. Von Sir Michael Fost. in “The Garden” besch.[rieben], [fl.], s.d., *W. Siehe s.n.* Exs. no. 22 (JE00022414 [digital image!], isolectotypes BM000958401!, E00332702!, LE00014023!, LE00014024!).—https://herbarium.univie.ac.at/database/detail.php?ID=457752 (accessed on 20 July 2021).

*Notes*—*Iris aschersonii* was described by Michael Foster from a dried plant and photographs received from Walter Erdmann Siehe [13]. Two images from the protologue refer to the original material of *I aschersonii.* The specimens from Siehe’s exsiccatum, accompanied by labels with the printed notes “Flora Orientalis” and “Ed. W. Siehe, Mersina”, may belong to the original material of *I. aschersonii*. As indicated on the labels attached to this exsiccatum, the plants were found in the Cilicia plain on limestone near Adana in 1898 and recognized as a new species by Foster in *The Garden*. Therefore, we believe that this exsiccatum and the dry plant indicated in the protologue refer to the same gathering, and are hence syntypes (see Art. 40, Note 1 of the ICN). As a consequence, the best-preserved specimen, i.e., JE00022414, is designated here as the lectotype. *Iris aschersonii* is endemic to Turkey, distributed in the Adana and Antalya provinces.

**Figure 1:**
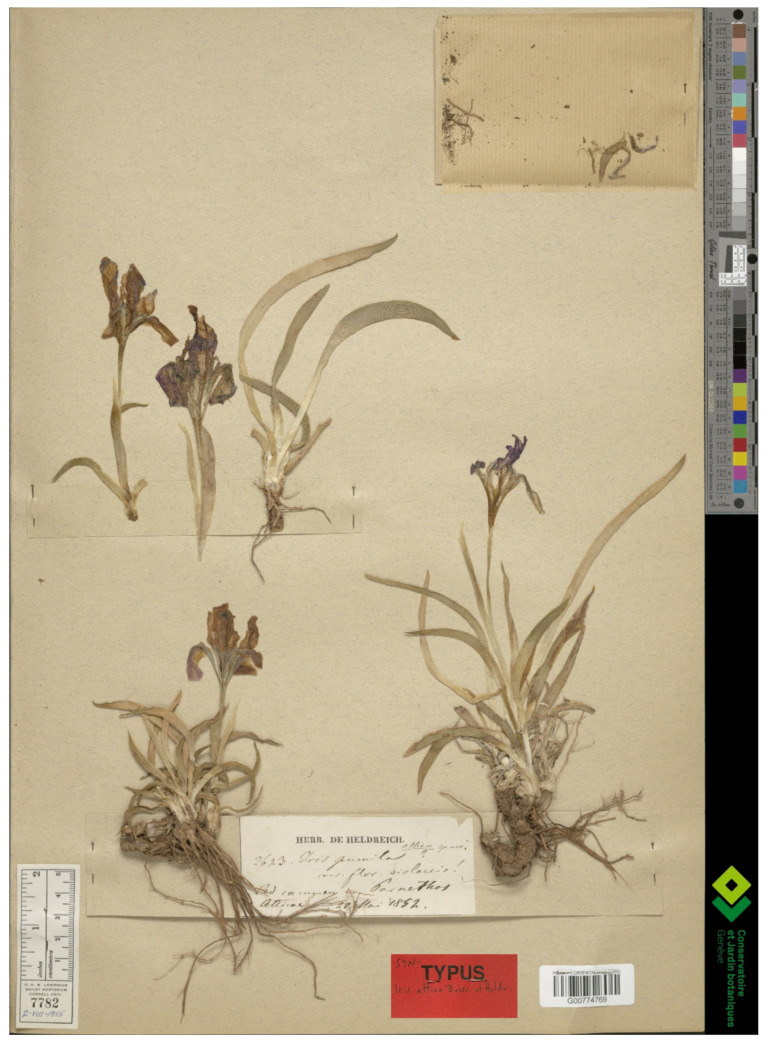
Lectotype of *Iris attica* (G00774769), by permission of the Curator.

(2) ***Iris attica*** Boiss. & Heldr. in Boiss., Diagn. Pl. Orient., ser. 2, 3(4): 91, 1859 ≡ *I. pumila* var. *attica* (Boiss. & Heldr.) Regel, Gartenfl. 11: 343, 1862 ≡ *I. pumila* subsp. *attica* (Boiss. & Heldr.) K.Richt., Pl. Eur. 1: 253, 1890.—Protologue citation: “Hab. in parte superiori montium Hymetti, Pentelici, Parnes Atticae Spruner! Boissier! Heldreich! in Parnassi regione inferiori Guicciardi!”.—Lectotype (designated here): [Greece, Central Greece Region], [Label with the printed note “Herb. De Heldreich”]: *Iris pumila* var. flor. violaceis! [*Iris*] *attica sp. nov*.? Ad cacumen m. Parnethos [Mount Parnassus] Atticae, [fl.], 20 May 1852, *Heldreich 2623*, Herb. Boissier (G00774769, isolectotypes P01844451! [two right-hand plants at the bottom], WAG1615963 [digital image!]).—Figure 1.—Syntypes: [Greece, Central Greece Region], [Label with the printed note “De Heldreich Flora Graeca Exciccata”]: *Iris attica* B. et H. In m. Parnassi reg., infer. pr. Rachova [Arachova], [fl.], April 1857, *J. Guicciardi s.n*. Exs. no. 1890, Herb. Boissier (G00774766 [digital image!]); *Iris attica*, Parnes, Attica, [fl.], May 1842, *Boissier s.n*. Herb. Boissier (G00774767 [digital image!]); [Label 1, handwritten by Boissier]: *Iris attica* v. *lutesuns*, [Label 2]: mons Parnes Attica, [fl.], May 1842, *Boissier s.n*. Herb. Boissier (G00774772 [digital image!]). [Attica Region]: *Iris lutescens* Lam. Gipfel des Pentelicon, [fl.], 1842, *Spruner s.n*. (G00379151 [digital image!]); *Iris lutescens* Lam. Pentelicon, Gipfel, [fl.], s.d., *Spruner s.n*. Herb. Boissier (G00774770 [digital image!]).

*Notes*—*Iris attica* was described by Pierre Edmond Boissier and Theodor Heinrich Hermann von Heldreich based on several gatherings collected by Boissier, Giacomo (or Jiacinto) Guicciardi-Barazetti, Theodor von Heldreich, and Wilhelm von Spruner in Greece [14]. The specimen from Boissier’s own herbarium (Figure 1) is designated here as lectotype because it matches the protologue and is the most informative one. It was collected near Mount Parnassus, central Greece, by Heldreich in 1852. *Iris attica* is close to *I. suaveolens* Boiss. & Reut., from which it differs by its longer perianth tube and bracteoles tightly sheathing the ovary and perianth tube. In Turkey, it occurs generally in western Anatolia (Balıkesir, Bilecik, Bolu, Çanakkale, Eskişehir, and Kütahya provinces) and has a fragmented distribution pattern (also see [4]).

(3) *Iris bornmuelleri* Hausskn., Flora 72(2): 141, 1889. = ***I. danfordiae*** (Baker) Boiss., Fl. Orient. 5(1): 124, 1882.—Protologue citation: “Habitat in saxosis subalpinis supra Amasiam Anatoliae borealis, flor. medio Mart”.—Lectotype (designated here): [Turkey, Amasya Province] *Iris bornmuelleri* m. Flora Anatol. bor., in saxos. subalpin. supra Amasiam, [fl.], 16 March 1889, *J. Bornmüller s.n.* (JE00020029 [digital image!]).—https://herbarium.univie.ac.at/database/detail.php?ID=457768 (accessed on 20 July 2021).—Other original material examined: [Turkey, Amasya Province] *Iris Bornmülleri* Hausskn. (*sp. nov*.), “*Flora*. 1889” (=*I. amasiana* Bornm. Möllers Gärtn. Zeit. 1889 IV in litt.), Amasia, in regionis subalpinae pascuis lapidosis (rara!), [c. 1200 m], [fl.], 11 March 1889, *J. Bornmüller s.n.* Exs. no. 2 (B100367900!, B100367901!, BM!, BR0000006884734 [digital image!], G!, K000499048!, LD1694046 [digital image!], LE00014042!, P02163247!).

*Notes*—*Iris bornmuelleri* was described by Heinrich Carl Haussknecht [15] from plants collected by Joseph Bornmüller. According to Haussknecht [15], Bornmüller gathered plants in the Amasya Province, northern Turkey, in mid-March 1889. The protologue of *I. bornmuelleri* cited the habitat and geographical data. However, it did not provide exact citation of specimens. Two gatherings collected by Bornmüller in the Amasya Province in mid-March 1889 were found. The specimen JE00020029 in Haussknecht’s own herbarium [16] matches the protologue of *I. bornmuelleri* and is designated as the lectotype. The specimens of the exsiccatum, annotated as “J. Bornmüller, Plantae Anatoliae Orientalis”, can also be referred to the original material of *I. bornmuelleri*. As it follows from the content of the printed labels, the plants of this exsiccatum were collected by Bornmüller in mid-March 1889 in the area from which *I. bornmuelleri* was described by Haussknecht. Baker [17] and Mathew [18] considered *I. bornmuelleri* as a taxonomic synonym of *I. danfordiae* (Baker) Boiss., which is correct.

(4) ***Iris histrio*** Rchb.f., Bot. Zeitung (Berlin) 30: 488, 1872 ≡ *Xiphion histrio* (Rchb.f.) Hook.f., Bot. Mag. 99: t. 6033, 1873 ≡ *Iris reticulata* var. *histrio* (Rchb.f.) Foster, Bulb. Irises: 57, 1892 ≡ *Iridodictyum histrio* (Rchb.f.) Rodion., Rod Iris – *Iris* L. (Vopr. Morfiol. Biol. Evol. i Sist.): 202, 1961.—Protologue citation: “Sommet du Liban, entre les deux mamelons appelès Taumets et Djerzine, fiès de Saïda”.—Lectotype (first step designated by Mathew [4] (p. 404), second step designated here): [Lebanon, South Governorate] Sommet du Liban, entre les deux mamelons appelés Taumates et Djerzîne, fiès de Saïda [Sidon], [fl.], 13 January 1854, *C. Gaillardot s.n.* Exs. no. 93 (K000499055!, isolectotypes G-BOIS00775177!, G-BOIS00775178!, K000499058!; P01793996!, P01840836!, P01840840!, P01844884!, P01847860!).—https://specimens.kew.org/herbarium/K000499055 (accessed on 20 July 2021).

*Notes*—*Iris histrio* was described by Heinrich Gustav Reichenbach, based on two cultivated plants and two specimens collected in Lebanon and belonging to the series “Herbier de Syrie” [19]. We were not able to locate the specimens examined by Reichenbach, but only duplicates of the specimens of the “Herbier de Syrie”, i.e., the no. 93, collected by Gaillardot in “Sommet du Liban, entre les deux mamelons appelès Taumets et Djerzine, fiès de Saïda”. We found these duplicates at G, K, and P. They are accompanied by labels with the printed note “No 93. HERBIER DE SERIE. 1855” and were initially identified as *I. reticulata* M.Bieb. In his work, Mathew [4] indicated “iso. K!” as the “type” of *I. histrio.* Thus, he actually designated the isosyntypes at K as the lectotype. However, we traced two isosyntypes at K (K000499055! & K000499058!). According to Art. 9.17 of the ICN, the type citation by Mathew [4] can be further narrowed to a single specimen by a second-step lectotypification. Therefore, K000499055!, which is more informative, is designated here as the lectotype of the name *I. histrio*. In Turkey, *I. histrio* is distributed in the Adana, Gaziantep, Hatay, Kahramanmaraş, Mersin, and Osmaniye provinces.

**Figure 2:**
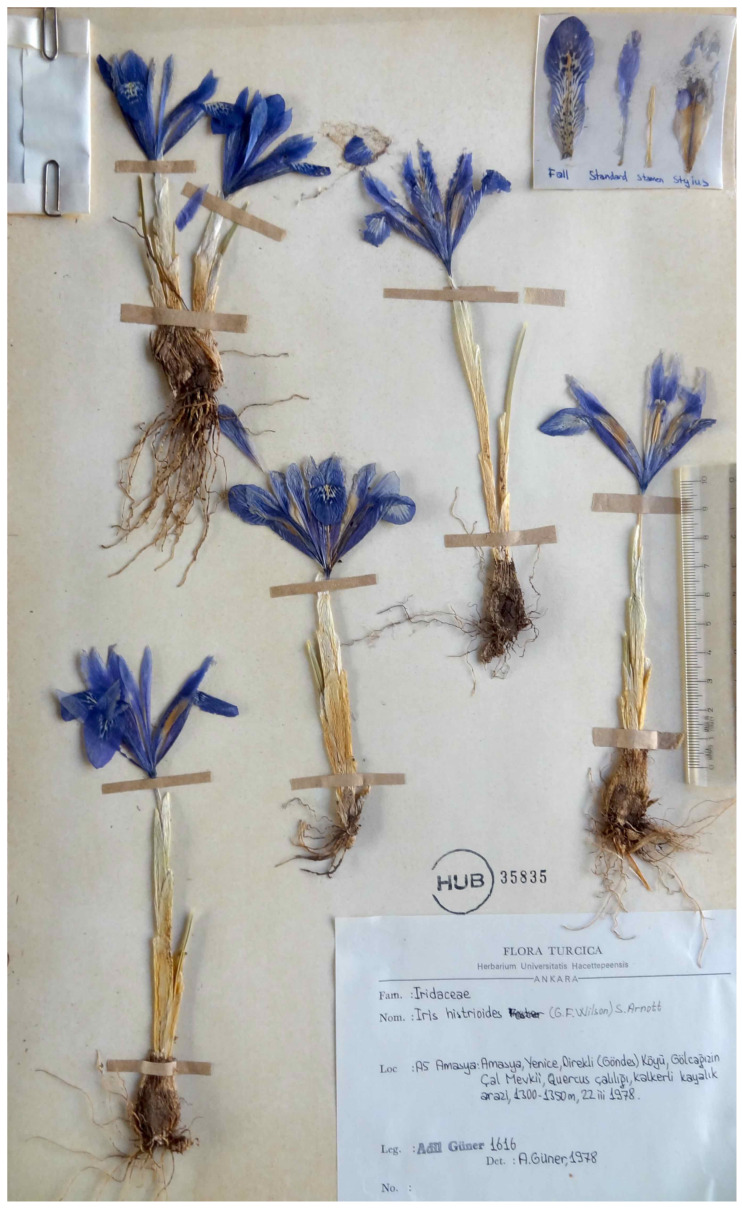
Neotype of *Iris histrioides* (HUB no. 35835), by permission of the Curator.

(5) ***Iris histrioides*** Reuthe, Gartenflora 40: 165, 1891.—“*Iridodictyum histrioides* (Reuthe) Rodion.”, Rod Iris – *Iris* L. (Vopr. Morfiol. Biol. Evol. i Sist.): 202, 1961, *nom. inval.* (Art. 41.5 of the ICN).—Protologue citation: [origin not specified].—Neotype (designated here): [Turkey, Amasya Province] Yenice, Direkli (Göndes) Köyü, Gölcağızınçal mevkii, meşe çalılığı, kalkerli kayalık arazi [Yenice District, Direkli Village, oak scrub, limestone rocky places], 1300–1350 m, 22 March 1978, *A. Güner 1616* [originally in Turkish] (HUB no. 35835!, isoepitypes AEF no. 7217!, ANK!, K001291623!).—Figure 2.

*Notes*—*Iris histrioides* was described by Gustavus Reuthe (Fox Hill Nursery, UK) from cultivated plants without indicating the collection locality [20]. The original material on which this name was based has not been found in the investigated herbaria. Therefore, a neotype may be selected according to the Art. 9.13 of the ICN. The neotype (Figure 2) is a complete specimen collected in Amasya Province, Turkey. It clearly shows the features of the inner perianth segments, the markings aggregated towards the centre of the lamina, which is characteristic of *I. histrioides*. *Iris histrioides* is an endemic to Turkey, distributed in the Amasya, Gümüşhane, Rize, Samsun, Sinop, and Tokat provinces (also see [10,21,22]).

**Figure 3:**
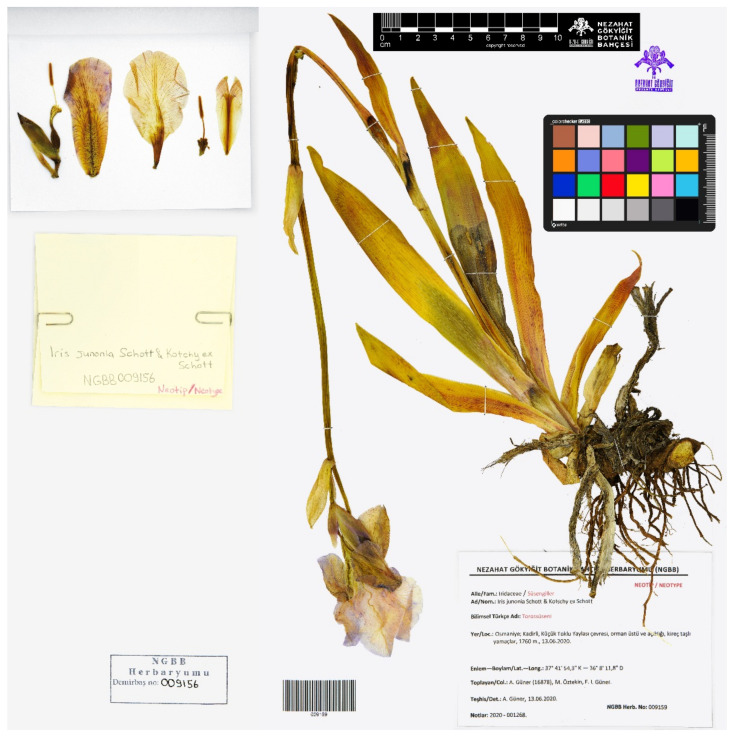
Neotype of *Iris junonia* (NGBB009159), by permission of the Curator.

(6) ***Iris junonia*** Schott & Kotschy ex Schott, Oesterr. Bot. Wochenbl. 4(26): 209, 1854 ≡ *I. pallida* var. *junonia* (Schott & Kotschy ex Schott) Baker, J. Linn. Soc., Bot. 16(91): 146, 1877.—Protologue citation: “Habitat in Tauro Ciliciae (Kotschy)”.—Neotype (designated here): [Turkey, Osmaniye Province] Osmaniye, Kadirli, Küçük Toklu Yaylası çevresi, orman üstü ve açıklığı, kireç taşlı yamaçlar, [Kadirli District, surroundings of Küçük Toklu, forest clearings or above timber line, limestone slopes], 37°41′54.3″ N 36°8′11.8″ E, 1760 m, [fl.], 13 June 2020, *A. Güner 16878, M. Öztekin & F.I. Güner* [originally in Turkish] (NGBB009159!).—Figure 3.

*Notes*—The name *Iris junonia* was validly published in a paper by Heinrich Wilhelm Schott and was ascribed there to Schott and Kotschy [23]. As follows from the protologue, the name was based on plants collected in the Cilicia region within the Taurus Mountains, southern Turkey, by Karl Georg Theodor Kotschy. According to Baytop [24], Kotschy botanized in the Taurus Mountains from June to early October 1853. Kotschy’s and Schott’s types are deposited at W, and some of the important material is in the Haynald Herbarium at BP [16,25]. Mathew [4] noted on the original material of *I. junonia* as follows: “holo. W – specimen not traced, perhaps destroyed”. The original material of *I. junonia* at W was destroyed by fire during World War II in 1943 (C. Bräuchler, pers. comm.), and no original material is now present at BP (I. Rácz, pers. comm.). Consequently, neotypification is required according to the Art. 19.11 of the ICN. For this purpose, we selected a specimen (Figure 3) collected in the Cilician Taurus Mountains, the region of Turkey from which the species had been described. *Iris junonia* is an endemic to Turkey, frequently found along the Taurus Mountains and in montane areas of western Anatolia in the Adana, Antalya, Bilecik, Burdur, Denizli, Isparta, Karaman, Kayseri, Kocaeli, Konya, Mersin, Muğla, Niğde, and Sakarya provinces. Currently, *I. junonia* is an accepted species, close to *I. germanica*.

(7) ***Iris masiae*** Leichtlin ex Foster, Garden (London 1871–1927) 61(1): 288, 1902 ≡ *Syrianthus masiae* (Leichtlin ex Foster) M.B.Crespo, Mart.-Azorín & Mavrodiev, Phytotaxa 232(1): 63, 2015.—“*Iris grant-duffii* subsp. *masiae* (Leichtlin ex Foster) Dykes“, Gen. *Iris*: 45, 1913, *nom. inval.* (see Art. 35.2, Ex. 6 of the ICN).—Protologue citation:—“Asia Minor”.—Neotype (designated here): [Turkey, Şanlıurfa Province] Süverek, in planitie ad Karadja Dagh, [fl.], 25 May 1888, *P. Sintenis 1219* (K001291625!, isoneotypes G00390262!, JE00020035, LD, P02163471!, WU0038645 & WU0038646 [digital images!]).—https://specimens.kew.org/herbarium/K001291625 (accessed on 20 July 2021).

*Notes*—*Iris masiae* was described by Foster [13] based on a cultivated plant originated presumably from Asia Minor. Foster stated that *I. masiae* is a purple-flowered relative of *I. aschersonii* and *I. grant-duffii* Baker. Dykes reported [26] that this species was discovered by Paul Sintenis in South Anatolia, Turkey, in 1888, and then introduced into cultivation by Max Leichtlin as follows: “an undetermined species gathered by Sintenis in the course of his journey in the East in 1888 on the steppes near the village of Süverek, on the lower slopes of the Karadja Dagh, a mountain which lies in the district of Diarbekr [Diyarbakır Province] in northern Mesopotamia”. We found duplicates of the same gathering made by Sintenis near Siverek, which is closer geographically to Diyarbakır, at G, JE, K, LD, P, and WU. According to the results of our research, no original material for *I. masiae* is known to exist. For this reason, K001291625! is here designated as neotype since it is the most informative. All the above-mentioned *I. masiae* isoneotypes are accompanied by labels with the printed note “P. Sintenis: Iter orientale 1888. Kurdistania. det. Dr. O. Stapf”, and the rest of the information was apparently handwritten by Stapf. In Turkey, *I. masiae* is distributed in the Diyarbakır, Gaziantep, Şanlıurfa, and Şırnak provinces. It comes nearest to *I. aschersonii*, from which it differs chiefly in the shape of the perianth segments [26].

**Figure 4:**
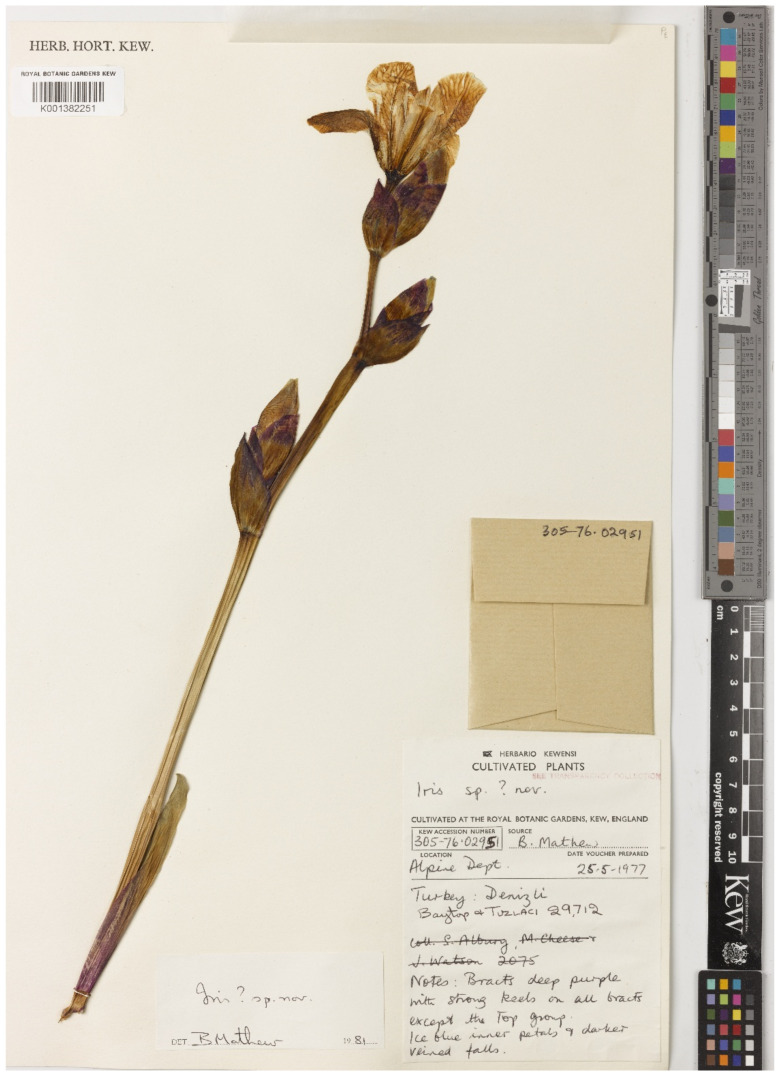
Lectotype of *Iris purpureobractea* (K001382251). Reproduced with the consent of the Royal Botanic Gardens, Kew.

(8) *Iris purpureobractea* B.Mathew & T.Baytop, Garden (London; 1975+) 107(11): 447, 1982, ***syn. nov*.** = ***I. junonia*** Schott & Kotschy ex Schott.—Protologue citation:—“Type: Turkey C2 Denizli, Honaz Dag, Kabardiç Yayla, 10 June 1973, A. Baytop & E. Tuzlaci ISTE 25725. Rhizomes of this collection flowered in Istanbul, 12 May 1974, and were given the number ISTE 29712”.—Lectotype (designated here): [Specimen from a cultivated plant no. 305-76.02951] *Iris sp*. ? *nov*. Turkey, Denizli, Baytop & Tuzlaci 29712; Notes: Bracts deep purple with strong keels on all bracts except the top group; ice blue inner petals and darker veined falls. [fl.], 25 May 1977, *B. Mathew s.n.* (K001382251!).—Figure 4.—Syntypes: [Specimen from a cultivated plant] *Iris* *purpureobractea* B.Mathew & T.Baytop, C2 Denizli, Honaz Dağı, Kabardiç yaylası, A. Baytop, ISTE 25725, cult. from rhizomes of type gathering, [fl.], s.d., *B. Mathew s.n*. (K!, possibly lost); [Turkey, Denizli Province] *Iris* *purpureobractea* B.Mathew & T.Baytop, C2 Denizli, Honaz Dağı, Kabardiç yaylası, [fl.], 10 June 1973, *A. Baytop & E. Tuzlacı 25725* (ISTE!).

*Notes*—*Iris purpureobractea* was described by Mathew and Turhan Baytop from plants collected on Mount Honaz, Denizli Province, southwestern Turkey [27]. The authors cited one gathering “ISTE 25725” and the cultivated plants (“ISTE 29712”) raised from the rhizomes of the original collection as “Type” of *I. purpureobractea*. As it follows from the protologue, the holotype was deposited at K and the isotype at ISTE. However, it remains unclear what part (a single specimen) of the type material was indicated by the authors as holotype. In 2014, when one of the co-authors of the present report (E.V.B.) visited Kew, the original material of *I. purpureobractea* was represented by at least two specimens cited above. One of them, mounted on a sheet bearing the number “ISTE 25725”, cited in the protologue, is currently absent from the Kew collection (A. Haigh, pers. comm.). Hence, the second specimen (Figure 4) is designated here as lectotype.

In the present study, *I. purpureobractea* is considered a synonym of *I. junonia* due to their morphological similarities. For example, in the natural populations of *I. purpureobractea*, yellow (as in *I. junonia*), blue, and purple forms are normally represented. Furthermore, not all individuals have purple bracts, which is a characteristic trait distinguishing *I. purpureobractea* from *I. junonia*. It was assumed [28,29] that *I. junonia* is intermediate between *I. pallida* Lam. and *I. germanica* L. or a hybrid of *I. pallida* and *I. variegata* L. [12]. A suggestion was also made that it has an ancient origin and is probably a form of *I. germanica* [12,30].

(9) *Iris reticulata* γ. [var.] *cyanea* Regel, Gartenflora 23: 162, 1874. = ***I. histrio*** Rchb.f.—Protologue citation: [origin not specified].—Lectotype (designated here): [illustration] “*Iris reticulata* M.B. γ. *cyanea*” in Regel [31] (t. 797, f. 1).—https://www.biodiversitylibrary.org/item/123793#page/225/mode/1up (accessed on 20 July 2021).

*Notes*—*Iris reticulata* var. *cyanea* was described by Eduard August von Regel from a cultivated plant without indicating the collection locality [31]. The protologue is accompanied by a colour drawing which is designated here as lectotype. This drawing is sufficiently detailed to provide a precise application of the name. The most characteristic diagnostic feature of *I. histrio* is that the lamina of the outer perianth segments is sparsely, however conspicuously blue blotched against the whitish background all over its surface. This diagnostic feature is clearly visible in the illustration published in the protologue of *I. reticulata* var. *cyanea*. Regel [31] pointed out that this variety is very similar to *I. histrio*. In our opinion, the two taxa are identical.

(10) *Iris reticulata* var. *histrioides* G.F.Wilson, Gard. Chron., ser. 3, 9: 117, 1891 ≡ *Iridodictyum histrioides* (G.F.Wilson) Nothdurft, Taxon 18(5): 600, 1969. = ***I. histrioides*** Reuthe.—Protologue citation: [origin not specified].—Neotype (designated here): [Turkey, Amasya Province] *Iris reticulata* M.B. var. *histrioides* (? hort.), floribus azureis non violaceis, Amasia: in regionibus alpinis et subalpinis, 16 March 1889, *J. Bornmüller s.n.* Exs. no. 3 (P01840835!, isoneotypes BAK! [except for the plant on the left of the sheet], BEI!, BM!, G! [2 specimens], K!, KFTA0003372!, LE! [4 specimens], M!, P02195571! [except for the plant in the middle of the sheet]).—https://science.mnhn.fr/institution/mnhn/collection/p/item/p01840835 (accessed on 20 July 2021).

*Notes*—*Iris reticulata* var. *histrioides* was described by George Fergusson Wilson from cultivated plants without indicating the collection locality [32]. In a brief description, Wilson specified that Leichtlin put these plants into commerce. Foster [22] (pp. 8–9) made the necessary explanations concerning the nomenclature history of this name. He noted that the plants had been sent to him before 1892 by Mary Wright from the American Mission in Amasia, and, since then, Leichtlin has obtained a large supply of such plants. Based on the fact that many specimens strongly resembled *I. histrio* in colour, Foster proposed to refer to it as “*Iris reticulata* var. *histrioides*”. Mathew [4] noted that the type specimen of the name had not been found. Our attempts to find the original material for this name in the framework of this study have not been successful either. The specimen designated here as a neotype for *I. reticulata* var. *histrioides* refers to Bornmüller’s exsiccatum “Pl. exs. Anatoliae orientalis a. 1889” collected in the Amasya Province, northern Turkey, where he carried out explorations in 1889 [24,33]. In his work, Bornmüller [33] noted that the iris he found was characterized by a beautiful colour, which varied a lot, and, therefore, could easily compete with that of *I. reticulata* (“sie zeichnet sich durch einen wundershönen Farbenschmuck, der viel variirt, aus, der sich leicht mit dem der iris messen kann”). The notes on the labels indicate that the colour of flowers in *I. reticulata* var. *histrioides* was azure (intense blue), which is a characteristic feature of *I. histrioides*.

**Figure 5:**
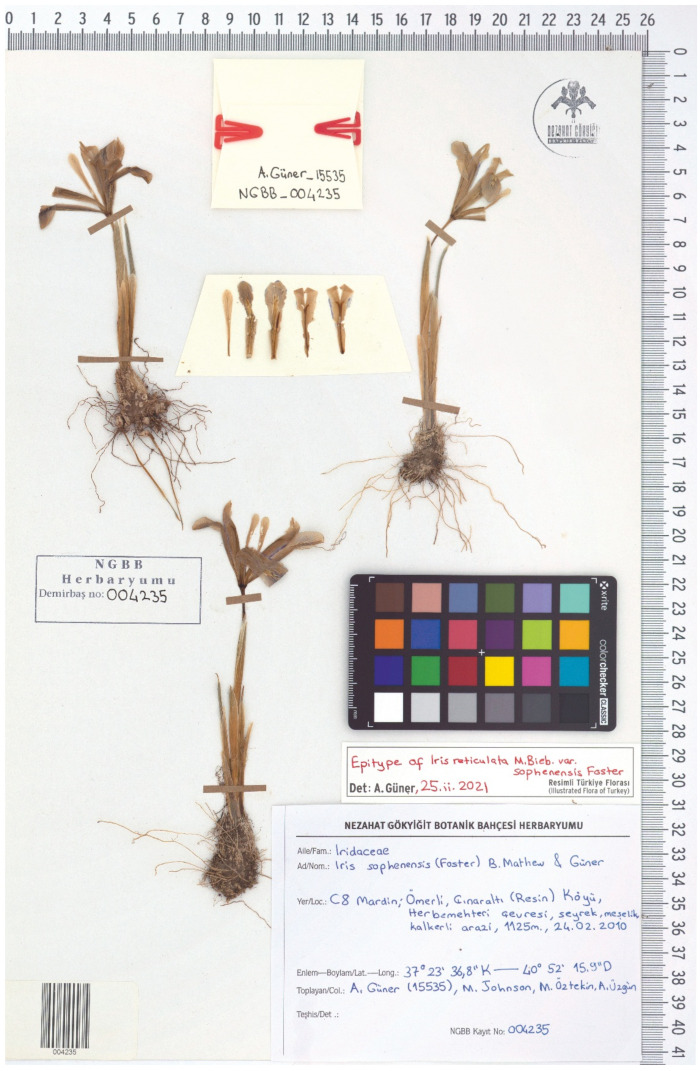
Epitype of *Iris reticulata* var. *sophenensis* (NGBB004235), by permission of the Curator.

(11) *Iris reticulata* var. *sophenensis* Foster, Gard. Chron., ser. 2, 23: 470, 1885 ≡ *I. histrioides* var. *sophenensis* (Foster) Dykes, Gen. *Iris*: 224, 1913 ≡ ***I. sophenensis*** (Foster) B.Mathew & Güner in Güner, Türk. Bitkileri List.: 539, 2012 ≡ *Iridodictyum sophenensis* (Foster) M.B. Crespo, Mart.-Azorín & Mavrodiev, Phytotaxa 232(1): 61, 2015.—Protologue citation: “… hills near Kharput”.—Lectotype (designated here): [Specimen from a cultivated plant], [M. Foster’s correspondence to Baker from Shelford]: […] *I. reticulata* var. … comes from Kharput [Harput], Asia Minor, [fl.], 15 February [1885], *M. Foster s.n.*; [Baker’s handwritings on a colour illustration and an envelope]: *Xiphium reticulatum* var., Kharput, Asia Minor, Prof. M. Foster, 16 February 1885 (K000499061!).—https://specimens.kew.org/herbarium/K000499061 (accessed on 20 July 2021).—Epitype (designated here):—TURKEY. [Mardin Province] Ömerli, Çınaraltı Village, around Herbemehteri, open oak wood, limestone, 1125 m, 37°23′36.8″ N 040°52′15.9″ E, 24 February 2010, *A. Güner 15535, M. Johnson, M. Öztekin & A. Özgün* [originally in Turkish] (NGBB004235!).—Figure 5.

*Notes*—*Iris reticulata* var. *sophenensis* was described and introduced into cultivation by Foster based on the plants from the bulbs sent by Mary E. Barnum from the American Mission at Harput (Elazığ Province, Turkey) in October 1884 [34]. These bulbs were gathered by Barnum in Sophene, the ancient name of the area around Harput. We found a specimen represented by a cultivated plant (K000499061!), which is the original material for the name. This sheet is accompanied by an envelope with dissected flowers and a colour illustration of the flower organs, together with Foster’s letter to J. Baker. However, it lacks a whole plant. Therefore, in order to avoid any ambiguity in the interpretation of the lectotype, an epitype is here designated (Figure 5). The selected epitype, is recently collected material from a locality geographically close to the type one, representing the same taxon as that to which the name was applied [22,28,35]. It provides additional characters that will contribute to the taxon identification. In the present study, this plant is treated as endemic to the Mardin Province, Turkey.

(12) ***Iris suaveolens*** Boiss. & Reut., in Boiss., Diagn. Pl. Orient., ser. 1, 2(13): 15, 1854.—Protologue citation: “Hab. in planitiebus Bulgariae propè Kustendje (Boiss.)”.—Lectotype (designated here):—[Specimen from a cultivated plant], [Label handwritten by G.F. Reuter]: *Iris pumila* L. var. Flores ochroleuci, petala exterior apice macula atroviolacea notate! Odor flores suavis … Jardin du Rivage; provenant de racines rapportées de la steppe de Kustendje par. m. E. Boissier e. 1842, [fl.], 22 April 1845, [*Reuter*] *s.n*. Herb. Boissier; [Label with the Reuter’s description]: *Iris suaveolens* B. et R. I. barbata, caule foliis breviori unifloro …; [Label handwritten by P.E. Boissier]: *Iris suaveolens* Boiss. et Reut. (G00164600 [digital image!]).—https://www.ville-ge.ch/musinfo/bd/cjb/chg/adetail.php?id=170119&lang=en (accessed on 20 July 2021).—Other original material examined: [Specimens from cultivated plants], [Labels handwritten by G.F. Reuter]: *Iris sp. nov.* fl. suaveolentes! Cult. in fenestra e radicib. ex ostiis Danubii ab amic. Boissier 1842 lectis, [fl.], s.d., [*Reuter*] *s.n.* Herb. Reuter (G00379146 [digital image!]); *Iris suaveolens* Boiss. et Reut. Cult. sur ma fenêtre, [fl.], March 1854, [*Reuter*] *s.n*. Herb. Reuter (G00379147 [digital image!]); *Iris suaveolens* Reut. m. Cult. sur la fenêtre, [fl.], March 1853, [*Reuter*] *s.n*. Herb. Reuter (G00379148 [digital image!]); *Iris pumila* L. var. Flores ochroleucus suave odoris, petalis exteriorib. macula atroviolacea notata, Jardin du Rivage, de bulbes de Kustendje, [fl.], 23 April 1845, [*Reuter*] *s.n*. Herb. Reuter (G00379150 [digital image!]). [Label handwritten by G.F. Reuter]: *Iris suaveolens* Reut. Cult. en vase, [fl.], February 1854, [*Reuter*] *s.n*.; [Label handwritten by P.E. Boissier]: *Iris suaveolens*. Herb. Boissier (G00379149 [digital image!]).

*Notes*—*Iris suaveolens* was described by Boissier and George François Reuter from plants cultivated at the Botanical Garden of Geneva, Switzerland [36]. These plants were raised from the rhizomes collected by Boissier near Constanța (formerly known as Küstendje), a city in the Northern Dobruja region, Romania. The specimen designated here as lectotype (G00164600) bears two labels containing Reuter’s handwritten [37] notes with a Latin description, which is consistent with the information provided in the protologue of *I. suaveolens*. The specimens from G are dated before May 1854, when *I. suaveolens* was published [38], and can also be considered the original material of this name. *Iris suaveolens* frequently grows in western and northern parts of Turkey, and differs from *I. attica* by its stem with 1–2 terminal flowers, equal in length, navicular, sharply keeled bracts and bracteoles, and ovary not tightly sheathed by bracteoles [4,5,39]. In Turkey, *I. suaveolens* is distributed in the Amasya, Ankara, Aydın, Balıkesir, Bilecik, Bursa, Çanakkale, Eskişehir, İstanbul, İzmir, Karabük, Kastamonu, Kırklareli, Kocaeli, Konya, Kütahya, Manisa, and Samsun provinces.

**Figure 6:**
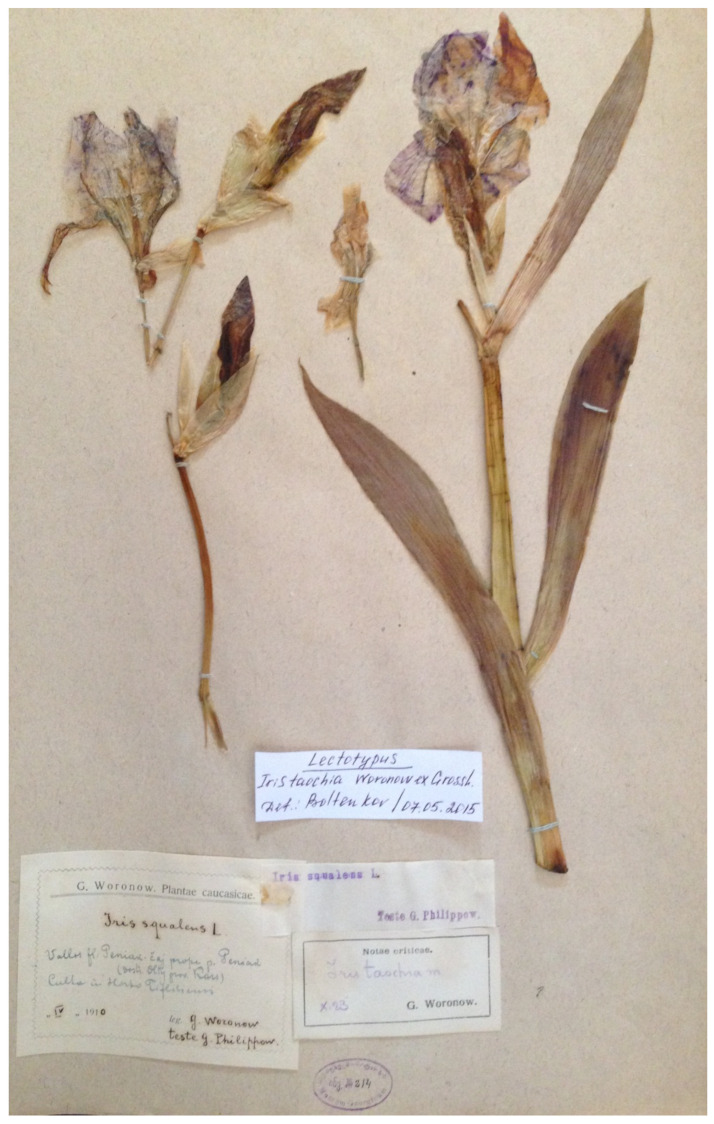
Lectotype of *Iris taochia* (TGM no. 214), by permission of the Curator.

(13) ***Iris taochia*** Woronow ex Grossh., Fl. Kavkaza 1: 256, 1928.—Protologue citation: “Turkey: Kars Oblast (Oltinsky Okrug) [originally in Russian]”.—Lectotype (designated here): [Specimen from a cultivated plant] *Iris squalens* L. Valles fl. Peniak-čaj, prope p. Peniak [Penek] (distr. Olty, prov. Kars), culta in Horto Tiflisiensis, [fl.], April 1910, *G. Woronow s.n.* (TGM no. 214!).—Figure 6.—Other original material examined: [Specimens from cultivated plants] *Iris squalens* L. Oltinsky Okrug, Kars Oblast, near Oltu (cultivated in Tiflis Botanical Garden), [fl.], April 1911, *G. Woronow s.n.* [originally in Russian] (TGM no. 213!); *Iris squalens* L. Culta in Horto Botanico Tiflisiensis (allatus ex Anzow [Anzav], distr. Olty, prov. Kars), [fl.], April 1910, *G. Woronow s.n.* (TGM no. 215!); *Iris squalens* L. Tiflis, Culta in Horto Botanico, Tubera e Kiasi-Këpri, distr. Olty, prov. Kars, [fl.], April 1910, *G. Woronow s.n.* (TGM no. 216!, TGM no. 217!).

*Notes*—*Iris taochia* was described by Alexander Alfonsovich Grossheim from cultivated plants that originated from Oltinsky Okrug of Kars Oblast, the Caucasus Viceroyalty of the Russian Empire in 1878–1917, which is part of today’s Erzurum Province, Turkey [40]. No specimen or gathering is indicated in the protologue. Grossheim ascribed the name *I. taochia* to Jurij (Georg) Woronow, a Russian botanist and plant collector. Since 1914, Woronow had worked at the Caucasus Museum (Tbilisi, Georgia) and, between 1921–1925, at the Tiflis Botanical Garden, except for a short period when he was engaged by the Botanical Museum of Academy of Sciences in St. Petersburg [41]. According to this information, we found Woronow’s specimens at TGM. All the selected specimens are accompanied by a printed label with the note “Notae criticae. G. Woronow”, on which Woronow handwrote “*Iris taochia* m. October [19]23”. While preparing his work [40], Grossheim was in Tbilisi [42] and used the TGM collection. Hence, the TGM specimens, in our opinion, can be considered the original material of the name *I. taochia*. One of them (Figure 6) is designated here as lectotype because it matches the protologue and is the most informative one. *Iris taochia* is a narrow endemic species to Erzurum Province, Turkey. In addition to Oltu District, it has been recorded from the vicinity of Tortum [4,39].

### 3.2. Remarks on the Previously Typified Names

(14) ***Iris bakeriana*** Foster, Bot. Mag. 115: t. 7084, 1889 ≡ *I. reticulata* var. *bakeriana* (Foster) B.Mathew & Wendelbo, Fl. Iranica 112: 43, 1975, *nom. illeg.* (Art. 53.1 of the ICN) ≡ *Iridodictyum bakerianum* (Foster) Rodion., Rod Iris – *Iris* L. (Vopr. Morfiol. Biol. Evol. i Sist.): 202, 1961.—Protologue citation: “… native of Armenia”.—Lectotype (designated by Wendelbo & Mathew [43] (p. 43), as “typus”): [Specimen from a cultivated plant], [Label 1, handwritten by M. Foster]: *I. bakeriana* sp. nov. Foster, Asia Minor, Armenia, near Mardin; [Label 2]: *Iris bakeriana* Foster! Type specimen of Bot. Mag. t. 7084! Asia Minor, near Mardin. From Prof. Michael Foster, Feb. 28, + March 19, 1889, sweetly scented (K000499060! [left-hand side specimen]).—https://specimens.kew.org/herbarium/K000499060 (accessed on 20 July 2021).

*Notes*—*Iris bakeriana* was described by Foster from the plants that had been raised from the bulbs sent by Frank Gates of the American Mission in Mardin in 1887 which flowered in cultivation in February–March 1889 [44]. The bulbs were gathered in south-eastern Turkey. In citing “Typus …: Armenia, Rev. G.F. Gates, cult. K!”, Wendelbo & Mathew [43] designated the specimen in K as the lectotype of *I. bakeriana* satisfying the requirements of Art. 7.11 of the ICN. At K, we found a single specimen (K000499060!) apparently related to the illustration from the protologue. In agreement with Mathew [18,21], we also accept *I. bakeriana*. In Turkey, it occurs only in the Batman and Mardin provinces. *Iris bakeriana* is very closely allied to *I. reticulata*, however differs chiefly in the nearly cylindrical, not tetragonal (a diagnostic character of *I. reticulata*), leaves [44].

**Figure 7:**
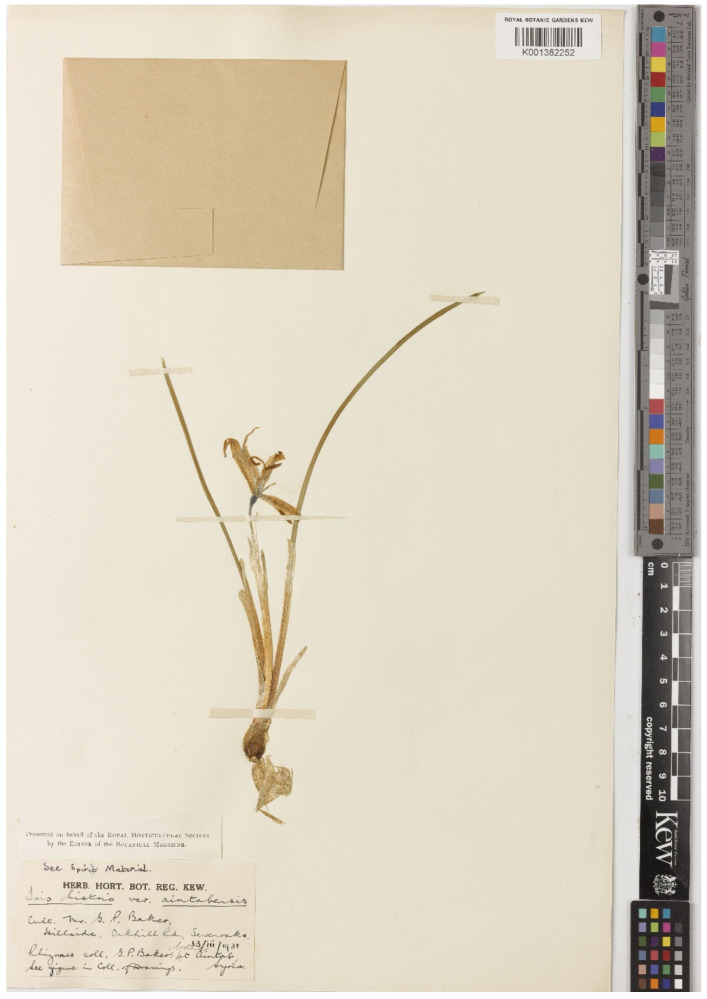
Lectotype of *Iris histrio* var. *aintabensis* (K001382252). Reproduced with the consent of the Royal Botanic Gardens, Kew.

(15) *Iris histrio* var. *aintabensis* G.P.Baker, Gard. Chron., ser. 3, 89: 137, 1931 ≡ *I. histrio* subsp. *aintabensis* (G.P.Baker) B.Mathew, Davis & Hedge, Festschrift: 97, 1989 ≡ *Iridodictyum aintabensis* (G.P.Baker) M.B.Crespo, Mart.-Azorín & Mavrodiev, Phytotaxa 232(1): 60, 2015 ≡ *Iris aintabensis* (G.P.Baker) Rukšāns, Int. Rock Gard. 112: 35, 2019. = ***I. histrio*** Rchb.f.—Protologue citation: [origin not specified].—Lectotype (indicated by Mathew [18] (p. 97), as “holo[type].”, corrected here): [Specimen from a cultivated plant], [Label 1]: *Iris histrio* var. *aintabensis*, Cult. Mr. G.P. Baker, Hillside, Oakhill rd., Sevenoaks [Kent, UK]; rhizomes coll. G.P. Baker at Aintab, Syria, [fl.], *G.P. Baker s.n*.; [Label 2]: Presented on behalf of the Royal Horticultural Society by the Editor of the *Botanical Magazine* (K001382252!).—Figure 7.

*Notes*—*Iris histrio* var. *aintabensis* was described by George Percival Baker without indicating the collection locality [45]. According to the protologue, this variety was exhibited at a Royal Horticultural Society show. Baker travelled to numerous mountain areas where he collected plants and subsequently introduced them into British gardens [46]. The epithet “*aintabensis*” is most likely derived from “Aīntāb” or “Antep”, the former (Ottoman) name for the modern city of Gaziantep located in the western part of the Southeastern Anatolia Region. This probably explains why Mathew [4,18,21] specified that the bulbs of *I. histrio* var. *aintabensis* had been collected near Gaziantep. Mathew [18] indicated that the holotype of *I. histrio* var. *aintabensis* was deposited at K as follows: “Type: S Turkey: a plant cultivated by G.P. Baker, exhibited at a Royal Horticultural Society Show, 10 Feb 1931, from bulbs collected near Gaziantep (holo. K!)”. We found a specimen from a cultivated plant in K which matches the information from Mathew’s work (Figure 7). This specimen was originally identified as “*Iris histrio* var. *aintabensis*”. It is possible that only a single specimen ever existed (which, in this case, would be the holotype), however, this cannot be established for certain because the name *I. histrio* var. *aintabensis* was published without a holotype. Therefore, a lectotype designation is required (Art. 9.3 of the ICN), and besides, the term “holotype”, used by Mathew [18], should be corrected to “lectotype” according to the Art. 9.10 of the ICN. Despite that Mathew [4,21] assigned this plant the taxonomic rank of variety, Crespo et al. [47] and Rukšāns [48] consider it a separate species. According to the protologue [45], the colours of the flowers in *I. histrio* var. *aintabensis* are characterized as Cambridge Blue with golden markings on the outer perianth segments. In our opinion, this name is a taxonomic synonym of *I. histrio*, a widespread species from southern Turkey. Adil Güner has studied *I. histrio* in natural populations over many years and has come to the conclusion that there are no morphological differences between *I. histrio* var. *aintabensis* and the autonymic variety, as the colour pattern of the blade is very variable within the species.

(16) *Iris musulmanica* Fomin, Vestn. Tiflissk. Bot. Sada 14: 46, 1909 ≡ ***I. spuria*** subsp. ***musulmanica*** (Fomin) Takht. in Takhtajan & Fedorov, Fl. Erevana, ed. 2: 330, 1972 ≡ *Xyridion musulmanicum* (Fomin) Rodion., Bot. Zhurn. (Moscow & Leningrad) 90(1): 58, 2005.—Protologue citation: “Habitat in humidis salsis prov. Elisabethpol, distr. Areschensis prope Nametabad-nour; in provincia Erivan, distr. Nachiczevan in salsis humidis prope stationem Davalu [Ararat]”.—Lectotype (designated by Fedtschenko [49] (p. 527), as “type”): [Armenia, Ararat Province] *Iris halophila* Pall. *musulmanica* Fomin, Davalu, Erivan Governorate, [fl.], 16 May 19[08], A*. Fomin s.n*. [originally in Russian] (TBI1025369! & TBI1025370!).—Syntypes: [Azerbaijan, Yevlakh Rayon] *Iris musulmanica sp. n.* Fomin, Elisabethpol Governorate, Areshsky Uyezd, Namet-Abadskij Nour, 17/30 May 1909, *Woronow & Schelkownikoff s.n.* [originally in Russian] (MHA!, TBI1025371!); Prov. Elisabethpol, distr. Aräsch, in pratis salsis prope Namet-Abadskij Nour inter Chaldan [Khaldan Village] et Aghdasch, 17/30 May 1908, [fl.], *A. Schelkownikow* & *G. Woronow s.n.* (LE01009783–LE01009785!).—https://psimg.jstor.org/fsi/img/size2/alukaplant/tbi/phase_01/tbi0009/tbi1025369.jpg (accessed on 20 July 2021).

*Notes*—*Iris musulmanica* was described by Alexander Vasiljevich Fomin from plants cultivated at the Tiflis Botanical Garden (Tbilisi, Georgia) [50]. They had been collected by him near Ararat village, known as Davalu, which, until 1935, was in western Armenia near the border with Turkey. Plants had also been collected by Woronow and Alexander Schelkownikow near Nemetabad village in the Yevlakh District, central Azerbaijan [51]. Thus, two gatherings were listed by the author in the protologue of *I. musulmanica*. Fedtschenko [49], indicating one of the gatherings, deposited at TBI as the “type” of *I. musulmanica* as follows: “Described from Armenia, Davalu. Type in Tiflis [originally in Russian]”. We checked the original material at TBI, which is located near the National Botanical Garden of Georgia where Fomin served as a director in 1902–1914 [51], and found two sheets related to the gathering from Davalu indicated by Fedtschenko [49]. Both sheets contain one plant in flower. One of the sheets (TBI1025369!) is accompanied by the original label with the printed note “Herbarium Horti Botanici Tiflisiensis” handwritten by Fomin. Similar information was handwritten by Liubov M. Kemularia-Nathadze on the label of the second sheet (TBI1025370!). Furthermore, both plants were initially mounted on a single herbarium sheet and, subsequently, one plant was removed from the sheet as indicated on the handwritten label by Kemularia-Nathadze: “This herbarium specimen was taken from a single herbarium sheet where another herbarium specimen remains; the label was copied, 16 October [19]42 [originally in Russian]”. Thus, the specimen TBI1025369! was the only one at TBI, and Fedtschenko’s indication should be accepted as the lectotype for *I. musulmanica*. Since the sheet TBI1025370! clearly labelled it as being part of a single specimen, it is a part of the lectotype and not a duplicate (Art. 8.3 of the ICN). The lectotype specimen was initially identified as *I. halophila* Pall. The second gathering is represented by at least five specimens, which are syntypes (Art. 40, Note 1 of the ICN). In Turkey, it occurs in the Ağrı, Bitlis, Erzincan, Erzurum, Hakkâri, Iğdır, Kars, Kayseri, and Van provinces.

(17) ***Iris reticulata*** M.Bieb., Fl. Taur.-Caucas. 1: 34, 1808 ≡ *Neubeckia reticulata* (M.Bieb.) Alef., Bot. Zeitung (Berlin) 21(40): 297, 1863 ≡ *Xiphion reticulatum* (M.Bieb.) Klatt, Linnaea 34: 572, 1866 ≡ *Iridodictyum reticulatum* (M.Bieb.) Rodion., Rod Iris – *Iris* L. (Vopr. Morfiol. Biol. Evol. i Sist.): 202, 1961.—“*Iris reticulata* var. *typica* Regel”, Trudy Imp. S.-Peterburgsk. Bot. Sada 2(2): 319, 1873, *nom. inval.* (Art. 24.3 of the ICN).—Protologue citation: “Habitat in Iberia. D. Adam”.—Lectotype (designated by Kuthatheladze [52] (p. 16), as “type”): [Georgia, Tbilisi] *Iris reticulata* m. Ex Iberia, Comm[unicavit]. Adam a[nno]. 1805, [fl.], [1800–1802], Adam *s.n.* Herb. Marschall von Bieberstein (LE01010451!).—https://data.rbge.org.uk/herb/E00373757 (accessed on 20 July 2021).—Other original material examined: [Georgia], [Label handwritten by Marschall von Bieberstein] *Iris reticulata*, Ex Iberia, [fl.], s.d., *Adam, Stewen s.n.* Herb. Marschall von Bieberstein (LE01011534!); [Label handwritten by Adams] *Iris reticulata* mihi, s.d., [fl.], *Adams s.n.* Herb. Willdenow (BW00987010 [digital image!]).

*Notes*—*Iris reticulata* was described by Friedrich August Marschall von Bieberstein from the plants collected by Johannes Michael Friedrich Adams, or Adam, in Georgia [53]. Adams collected plants in the Georgian regions (Kartli, Kakheti, and Somcheti) during the expedition of Count Apollo Mussin-Pushkin in 1800–1802 [54]. Kuthatheladze [52] indicated that the specimen represented by two plants and containing labels with handwritings made by Marschall von Bieberstein (“*Iris reticulata* m. Ex Iberia, Comm. Adam a. 1805”) and Alexander Grossheim (“Typus!”), deposited at LE, is the type of *I. reticulata*. The content of the label on this specimen (LE01010451!) matches the protologue and indicates that it was obtained by Marschall von Bieberstein from Adams in 1805 [55]. Thus, the specimen from Marschall von Bieberstein’s herbarium (LE01010451!) refers to the original material of *I. reticulata*. Mathew [21] indicated that the type of *I. reticulata* was deposited at LE as follows “Type: USSR, Caucasus "Iberia", without a precise locality, *Adam* (holo. LE)”. However, the Kuthatheladze’s designation of a type has priority and should be accepted as lectotype (Art. 7.11 of the ICN). The specimens from the personal herbaria of Marschall von Bieberstein (LE01011534!) and Carl Ludwig Willdenow (BW0098701) can also be referred to the original material of *I. reticulata*, as they were gathered by Adams. In Turkey, *I. reticulata* is distributed in the Kahramanmaraş, Kars, Kayseri, Malatya, Muş, Sivas, Şanlıurfa, Tunceli, and Van provinces.

**Figure 8:**
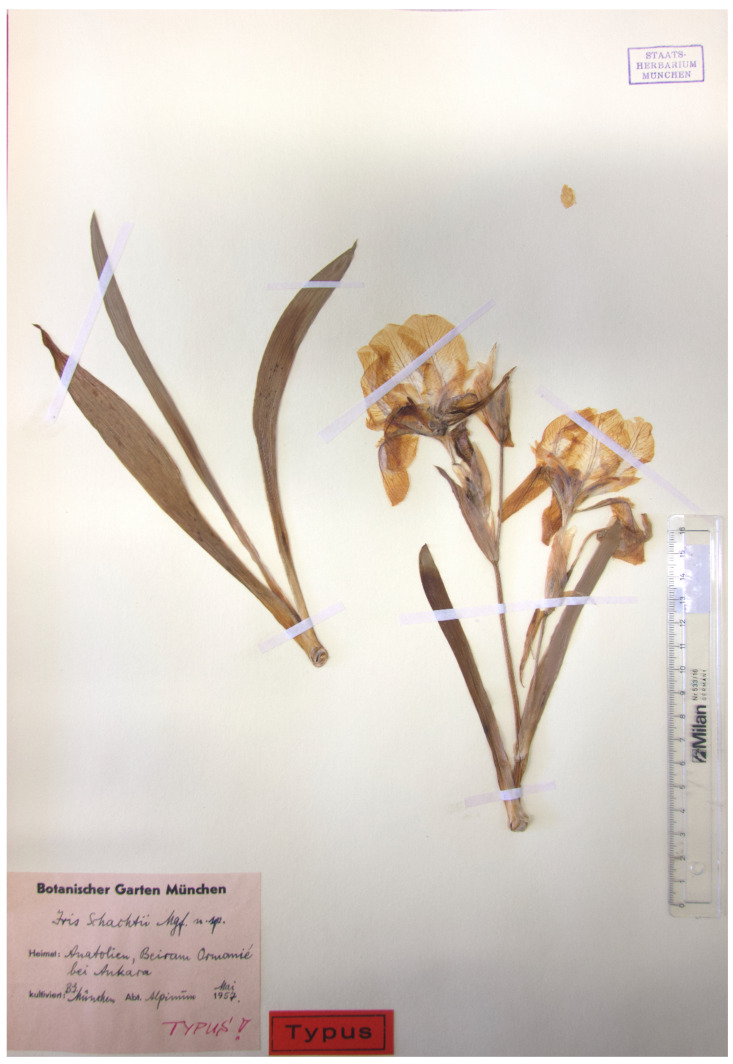
Lectotype of *Iris schachtii* (M), by permission of the Curator.

(18) ***Iris schachtii*** Markgr., Gartenbauwissenschaft 22(4): 550, 1957.—Protologue citation: “Anatolien: Beiram-Ormanié, 50 km südöstlich von Ankara, in trockener Staudenflur häufig, leg. W. Schacht, blühend in Botanischen Garten München, Mai 1957”.—Lectotype (indicated by Mathew [4] (p. 395), as “holo.”, corrected here): [Specimen from a cultivated plant] *Iris schachtii* Mgf. n. sp. [Native]: Anatolien, Beiram-Ormanié [Beynam Forest] bei Ankara; [cultivated] B.G. München, Alpinūm, [fl.], May 1957, *s.coll. s.n.* (M!).—Figure 8.

*Notes*—*Iris schachtii* was described by Friedrich Markgraf from plants cultivated at the Munich Botanical Garden [56]. These plants were collected in the Beynam Forest near Ankara (Turkey) by Wilhelm Schacht, after whom the species was named. Mathew [4] indicated the specimen from a cultivated plant at M as “holotype” (Figure 8). Hence, according to the Art. 9.10 of the ICN, the term “holotype”, used by Mathew [4], should be corrected to “lectotype”. *Iris schachtii* is an endemic to Turkey, distributed in the Kırşehir, Konya, Malatya, Sivas, Uşak, and Yozgat provinces.

(19) *Xiphion danfordiae* Baker, J. Bot. 14: 265, 1876 ≡ ***Iris danfordiae*** (Baker) Boiss., Fl. Orient. 5(1): 124, 1882 ≡ *Juno danfordiae* (Baker) Klatt, Abh. Naturf. Ges. Halle 15(3/4): 362, 1882 ≡ *Iridodictyum danfordiae* (Baker) Rodion., Rod Iris – *Iris* L. (Vopr. Morfiol. Biol. Evol. i Sist.): 202, 1961.—Protologue citation: “Mrs. Danford … in the spring of the present year [1876] … the Cilician Taurus”.—Lectotype (indicated by Mathew [4] (p. 405), as “holo.”, corrected here): [Turkey, Adana Province] *Xiphion Danfordiae* Baker, n. sp., Cilician Taurus, recd. 6/[18]76, [fl.], [March 1876], Mrs *A.E. Danford s.n.* (K000499051!).—http://specimens.kew.org/herbarium/K000499051 (accessed on 20 July 2021).

*Notes*—*Xiphion danfordiae* was described by Baker [57] from plants gathered by Antoinette Emily Dyce, or Mrs Danford [58], in 1876 in the Taurus Mountain Range in southern Anatolia, Turkey (see also [59] (p. 268)). The protologue is accompanied by Mrs Danford’s letter in which she indicated that the specimens had been found on the 24th of March twice, on “the northern side of the Anaxlia Mountain” and “near the village of Anascha” at an elevation of 4000 ft. In the Kew Herbarium, we found a specimen (K000499051!) containing parts of five plants, a pencil drawing of plants in their habitat, and Mrs Danford’s letter. Mathew [4] provided the information taken from the protologue of *X. danfordiae* and indicated that the holotype was deposited at K as follows: “(holo. K)”. As the protologue citation does not refer to a single specimen, the term “holotype”, used by Mathew [4] (p. 405), should be corrected to “lectotype” according to the Art. 9.10 of the ICN. *Iris danfordiae* is a species endemic to Turkey, distributed in the Adana, Amasya, Erzincan, Gümüşhane, Malatya, Niğde, Ordu, and Sivas provinces. The yellow flowers and the reduced inner perianth segment clearly distinguish this species from all other Reticulata species.

## 4. Conclusions

In Turkey, *Iris* s.l. is one of the richest genera in terms of number of species. The present contribution is a part of the full taxonomic revision of the genus for the Turkish flora carried out by A. Güner for *Resimli Türkiye Florasi* (*The Illustrated Flora of Turkey*) and a part of E.V. Boltenkov’s continuing research on the *Iris* taxonomy, aiming to update the nomenclature and extend the existing systematic knowledge. We have summarized the previously published typifications for some names and designated the lectotypes/neotypes for the thirteen untypified names. Furthermore, a study of the original material and field observations on *I. purpureobractea* have shown it to be a synonym of *I. junonia*.
